# Benchmarking tocilizumab use for giant cell arteritis

**DOI:** 10.1093/rap/rkac037

**Published:** 2022-05-09

**Authors:** Richard Conway, Michael S Putman, Sarah L Mackie

**Affiliations:** 1 Department of Rheumatology, St. James’s Hospital; 2 Clinical Medicine, Trinity College Dublin, Dublin, Ireland; 3 Medical College of Wisconsin, Milwaukee, WI, USA; 4 Leeds Institute of Rheumatic and Musculoskeletal Medicine, University of Leeds; 5 NIHR Leeds Biomedical Research Unit, Leeds Teaching Hospitals NHS Trust, Leeds, UK

GCA is a common condition, with a lifetime risk in Northern European populations of 1% for women and 0.5% for men [1]. The landmark Giant-Cell Arteritis Actemra (GiACTA) trial revolutionized the treatment of GCA, which for 70 years had been a disease primarily treated with glucocorticoid monotherapy [2]. After GiACTA, the US Food and Drug Administration and the European Medicines Agency approved tocilizumab as a treatment for GCA. The approved Roche summary of product characteristics (SPC) states that ‘treatment beyond 52 weeks should be guided by disease activity, physician discretion, and patient choice’ [3]. Subsequently, EULAR guidelines recommended the use of tocilizumab in people with GCA with relapsing disease or at high risk of glucocorticoid-related adverse events [4].

The UK National Institute of Health and Clinical Excellence (NICE) Technology Appraisal TA518 recommended tocilizumab as an option for GCA ‘only if they have relapsing or refractory disease, they have not already had tocilizumab, and tocilizumab is stopped after 1 year of uninterrupted treatment at most’ [5]. National Health Service (NHS) England adopted these criteria via a central commissioning process for ‘high-cost drugs’, ensuring physician adherence. The 1 year rule specific to GCA is included in the current tocilizumab patient information leaflet from the UK charity Versus Arthritis (last revised April 2021) [6]. During the coronavirus disease 2019 (COVID-19) pandemic, NHS England produced a rapid policy document allowing extended prescribing of tocilizumab for GCA beyond 1 year for certain GCA subgroups perceived as higher risk; this special provision ended in April 2022 [7].

NICE covers only England and Wales; guidance for Scotland is issued by the Scottish Medicines Consortium (SMC). The SMC did not restrict tocilizumab to relapsing or refractory GCA and, although mentioning a 12 month ‘clinical stopping rule’, also repeated verbatim from the SPC the statement about treatment beyond 52 weeks [8]. This is similar to the clinical practice guideline issued more recently by ACR, which essentially leaves the decision to start tocilizumab up to the patient and their treating physician [9].

It is in this context that Cronin *et al.* [10] report real-world data on tocilizumab utilization and efficacy in people with GCA in this issue of *Rheumatology Advances in Practice*.

In line with EULAR guidelines, but not the more restrictive NICE guidance for England and Wales, the authors adopt a position that those with baseline glucocorticoid risk factors and those with relapsing GCA should be considered for tocilizumab. Nineteen out of 63 (30%) of their Scottish GCA cohort were prescribed tocilizumab. Most had relapsing or refractory GCA; 63% of their patients had baseline glucocorticoid risk factors. Eleven per cent were prescribed tocilizumab at GCA inception owing to risk of CS side effects. The authors opine that their data might reflect hesitancy and an under-utilization of tocilizumab in their cohort.

Lower than expected use of tocilizumab does not necessarily imply under-utilization. Risk aversion inevitably influences medical decision-making; risks are arguably greater for biologics and among elderly patients [11, 12]. Efforts to be transparent about side-effects of new medications have resulted in a plethora of print and online information describing the potential adverse effects of biologic drugs, including tocilizumab [6]. High-dose or long-term glucocorticoid treatment carries significant, well-known risks, which historically might have been framed differently when there was no other treatment option. Attitudes are changing now that new therapeutic regimens for many diseases offer the possibility of lower doses or shorter durations of glucocorticoid, but updates to patient educational materials might be slower to reflect these changing attitudes. The context of this study, which took place during the COVID-19 pandemic, should also be considered. Real or perceived risk of shortages of tocilizumab might have influenced both patient and physician willingness to initiate. Finally, the study covers a period not long after tocilizumab approval for GCA; it is natural that there will be an initial slow uptake in use with any new treatment, and we might not be seeing a true plateau in utilization rates reflected in these results.

Many GCA patients, moreover, might not be candidates for tocilizumab owing to relative contraindications. Most notably, physicians might be reluctant to commence tocilizumab in patients with a history of diverticular disease, which increases with age and affects up to half of patients aged >50 years. Physicians might also avoid tocilizumab in patients with a history of recurrent infections, although it should be noted that patients in GiACTA who received tocilizumab had fewer infectious complications than those in the glucocorticoid-only arm of the study. Injections are off-putting for some patients, although in our experience many patients become comfortable with this over time. Long-term data on tocilizumab effects in the oldest old patients with GCA are not yet available, and caution in such populations might be warranted.

What proportion of people with GCA should receive tocilizumab? From our personal experience, the use of tocilizumab described here is greater than that in England, but similar to what has been seen in the USA and Ireland, although rates in these countries have yet to be characterized fully. In April 2021, NHS England reported that only 250 patients in England were taking tocilizumab for GCA [7]. Although EULAR guidelines recommend tocilizumab for the subset of GCA patients at high risk of glucocorticoid-related adverse events, defining this group might be challenging. Hypertension was the most common risk factor in this cohort, but is well-controlled hypertension alone sufficient to justify the addition of a biologic therapy at GCA diagnosis? Age or other co-morbidities, such as osteoporosis or prior cardiovascular disease, might be seen as more compelling reasons. Arguably, patients who might benefit the most, including those with multimorbidity, polypharmacy and difficult life circumstances, might be least likely to receive tocilizumab. The development and validation of a risk factor-based model might help with some of these difficult decisions, alongside better assessment of glucocorticoid toxicity in clinical practice [13].

The alternative approach of offering tocilizumab to all patients with GCA, which was endorsed by the ACR, introduces a different set of problems [9]. Like many biological agents, tocilizumab is expensive; leaving the decision to individual prescribers, without guidance on risk stratification, is likely to lead to substantial variation in treatment costs and clinical outcomes. Furthermore, because not all patients were eligible for the GiACTA study, its findings might not be generalizable to all patients with GCA, including some important GCA phenotypes. Exclusion from GiACTA of patients at highest risk (those who had received intravenous glucocorticoids) has limited our understanding of the efficacy of tocilizumab for patients presenting with critical visual ischaemia.

Perhaps most importantly of all, it is still difficult to predict at diagnosis of GCA how an individual patient will respond to glucocorticoid or tocilizumab treatment over time with regard to important outcomes, including relapse/remission, treatment toxicity and disease-related complications. Real-world experience with tocilizumab will probably inform us further, particularly the natural experiment that is the result of the reimbursement decisions of individual jurisdictions, resulting in very different practice patterns even in adjacent geographical locations. Meanwhile, we must do our best with the data we have, recognizing that decisions about tocilizumab prescribing in GCA are not always easy and involving our patients in making these decisions to the degree that they desire.

*Funding:* No funding was received for this manuscript.

*Disclosure statement:* R.C.: Speakers bureau: Janssen, Roche, Sanofi, Abbvie. Research funding related to clinical trials by Abbvie (CONTExT-RA, UPHOLD, SENSE). Research funding from Janssen (unrestricted education grant).

M.S.P. receives research funding related to clinical trials by Abbvie (SELECT-GCA) and AstraZeneca (MANDARA) and consulting payments from Novartis.

S.L.M.’s institution received research funding related to clinical trials from Sanofi (sarilumab in GCA; sarilumab in PMR), GSK (sirukumab in GCA) and from Roche for an observational tocilizumab registry. S.L.M. conducted consulting work on behalf of her institution for Roche, Sanofi, AbbVie and AstraZeneca, and gave non-promotional educational talks for Chugai, Pfizer and Vifor; all fees were paid to her institution, and she did not receive any personal fee. S.L.M. was supported by Roche to attend EULAR2019 and by Pfizer to attend ACR Convergence 21 virtually. S.L.M. is a Patron of the charity PMRGCAuk.

## Data availability statement

No new data are presented in this manuscript.
